# Electrospun PVDF-Based Polymers for Lithium-Ion Battery Separators: A Review

**DOI:** 10.3390/polym16202895

**Published:** 2024-10-14

**Authors:** Juanxia He, Lihong Yang, Xingzhe Ruan, Zechun Liu, Kezhang Liao, Qingshan Duan, Yongzhong Zhan

**Affiliations:** 1 School of Resources, Environment and Materials, Guangxi University, Nanning 530004, China; hejuanxia2005@126.com (J.H.); a2503215889@163.com (L.Y.); 2215391087@st.gxu.edu.cn (X.R.); 2315301037@st.gxu.edu.cn (Z.L.); 19152663240@163.com (K.L.); 2 State Key Laboratory of Featured Metal Materials and Life-Cycle Safety for Composite Structures, Nanning 530004, China; 3 School of Light Industry and Food Engineering, Guangxi University, Nanning 530004, China; qs_duan@gxu.edu.cn

**Keywords:** electrospinning, polyvinylidene fluoride, lithium-ion batteries, separators, influencing factors

## Abstract

Lithium-ion batteries (LIBs) have been widely applied in electronic communication, transportation, aerospace, and other fields, among which separators are vital for their electrochemical stability and safety. Electrospun polyvinylidene fluoride (PVDF)-based separators have a large specific surface area, high porosity, and remarkable thermal stability, which significantly enhances the electrochemistry and safety of LIBs. First, this paper reviewed recent research hotspots and processes of electrospun PVDF-based LIB separators; then, their pivotal parameters influencing morphology, structures, and properties of separators, especially in the process of electrospinning solution preparation, electrospinning process, and post-treatment methods were summarized. Finally, the challenges of PVDF-based LIB separators were proposed and discussed, which paved the way for the application of electrospun PVDF-based separators in LIBs and the development of LIBs with high electrochemistry and security.

## 1. Introduction

Lithium-ion batteries (LIBs) (Table A1: all abbreviations with their explanations) have the advantages of superior energy density, long cycle life, and no memory effect [1,2,3,4,5]; thus, they have been extensively used in portable electronic products, new energy vehicles, civil aircraft, and so on [6,7,8,9]. In addition, as a novel energy source, LIBs also contribute to alleviating the environmental pollution and energy crisis caused by the depletion of traditional energy and achieving the goal of carbon neutrality [10,11,12,13].

Separators are key parts of LIBs. On the one hand, they promote the free flow of lithium-ions (Li^+^) via accommodating the electrolyte and ensuring the electrochemical performance of LIBs; on the other hand, as physical isolation, they are the last barriers to avoid the contact reaction of positive and negative electrodes [3,14,15,16,17,18,19]. Tri-layered polyolefin separators include a polyethylene (PE) inner layer and two polypropylene (PP) outer layers, which are commonly used in LIBs due to chemical inertia and safety. However, commercial polyolefin separators with poor electrolyte wettability limit Li^+^ transmission, and the electrolyte quickly spills and ignites in the case of depressurization or puncture, which hinders the development of high-performance LIBs [20,21,22]. Additionally, their low melting point frequently results in thermal runaway due to thermal, electrical, and mechanical abuse [18,23,24,25,26], which easily causes LIBs’ combustion and explosion accidents [27,28]. Therefore, it is crucial to develop separators with outstanding wettability and heat resistance to enhance LIBs’ energy density and safety.

**Figure 1:**
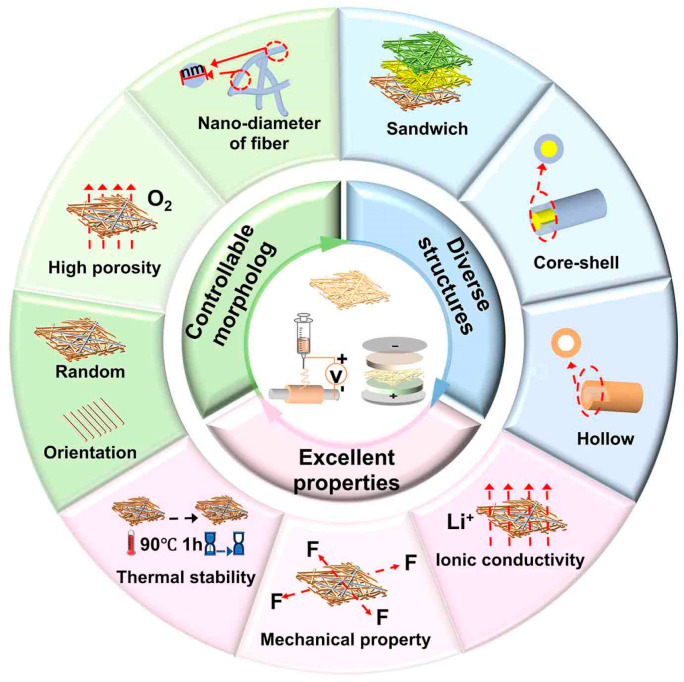
Features of electrospun PVDF-based LIB separators.

Poly(vinylidene fluoride) (PVDF) and its polymers have the characteristics of non-toxicity, thermotolerance, and high dielectric constant, which is a potential and promising candidate for polyolefin separators of LIBs [29,30,31,32]. These approaches for preparing LIB separators can be divided into three ways: template synthesis [4]; phase separation [2,33]; and electrospinning [34,35,36,37]. Among them, electrospinning not only has the advantages of simple operation, low cost, and high efficiency but is also an essential technology for the continuous preparation of nanofiber separators with large specific surface areas and high porosity [36,38,39,40,41,42]. Electrospun PVDF-based separators also have characteristics of controllable morphology, diverse structure, and excellent properties (such as thermal stability), which can concurrently solve the main issues of poor wetting and safety of commercial polyolefin separators when they are applied in LIBs (Figure 1). [43,44,45,46]. Although the application of electrospun PVDF-based separators in LIBs has been extensively investigated, the lack of systematic study on the effects of various factors in the electrospinning process and post-treatment methods about morphology, structures, and performance of electrospun PVDF-based separators has hindered further improvement of performance and application in LIBs.

Firstly, the brief timeline of electrospun PVDF-based LIB separators was summarized. Secondly, based on research on electrospun PVDF-based LIB separators and their entire preparation process of electrospinning solution, electrospinning, and post-processing, their key parameters concerning morphology, structures, and properties were systematically analyzed and discussed. Finally, the current research challenges of PVDF-based LIB separators were proposed, which provide an effective reference for high-performance PVDF-based separators as well as LIBs.

## 2. Electrospun PVDF-Based LIB Separators

In order to ensure LIBs’ normal operation, separators should meet the relevant basic requirements, including feature parameters (thickness, pore size, porosity, electrolyte contact angle, and uptake) and performance parameters (ionic conductivity, tensile strength, and thermal stability) [3,14,47]. Moreover, a lot of academics have conducted extensive research on electrospun PVDF separators in LIBs, which have been developed for more than 20 years [48,49,50,51,52,53].

### 2.1. Basic Requirements of the LIB Separators’ Key Parameters

The electrospun PVDF-based separators in LIBs match the basic requirements (Table 1), including chemical and electrochemical stability, namely, non-reactivity with the internal materials [3,14,47].

These parameters involve feature and performance parameters, and the former includes thickness, pore size, porosity, electrolyte contact angle, and uptake. Firstly, the thinner the separator, the smaller the LIBs’ internal resistance, and the higher the energy density [3]. Secondly, the uniform thickness promotes the distribution of the internal current in LIBs, which reduces the short circuit caused by the excessive local current [3,47]. The large-scale size and uneven structure of pores on separators may cause the LIBs’ electrode materials to shuttle through pores, which will cause a “shuttle effect” and make a potential hazard [3,47]. As a result, separators used in LIBs with high porosity and small electrolyte contact angles have the characteristics of high electrolyte uptake, low internal resistance, high ionic conductivity, and power density [47,54].

Feature parameters affect the performance parameters of separators, namely, ionic conductivity (containing electrolyte), tensile strength, and thermal stability [3]. The ionic conductivity of separators containing electrolytes is usually in the range of 10^−3^ to 10^−1^ S cm^−1^, which increases when pore size, porosity, electrolyte contact angle, and uptake rise synchronously or non-synchronously [55,56]. And the larger the values, the better the performance of LIBs [3,57]. In addition, the tensile strength of separators is mainly negatively correlated with their thickness, pore size, and porosity [3,45,47]. The greater the tensile strength, the better the LIBs’s safety [3,45,47]. Although thermal stability is primarily determined by the inherent material properties, separators with high porosity are more likely to shrink due to high temperatures, which increases the possibility of unsafe accidents during their service [47,58].

**Table 1:** Basic requirements for key parameters of LIB separators.

Key Parameters		Basic Requirements	Related Formulas	Ref.
Feature parameters	Thickness.	<25 μm	-	[47]
	Pore size	<1 μm	-	[1]
	Porosity.	>40%	$Porosity(\%)=\frac{W-W_{0}}{\rho_{L}V_{0}}\times 100\%$ ^1^	[56]
	Electrolyte contact angle	The smaller, the better	-	[54]
	Electrolyte uptake	-	$Electrolyte-uptake(\%)=\frac{M-M_{0}}{M_{0}}\times 100\%$ ^2^	[59]
Performance parameters	Ionic conductivity	-	$\sigma =\frac{D}{R_{b}A}$ ^3^	[56]
	Tensile strength	>98.06 MPa		[46]
	Thermal stability	Shrinkage rate < 5% (90 °C for 1 h)	$Thermal-shrinkage(\%)=\frac{A_{0}-A}{A_{0}}\times 100\%$ ^4^	[21]

^1^ *W*_0_, *W*—the mass of the separator before and after absorbing n-butanol, kg; ρ_L_—n-butanol density, kg m^−3^; *V*_0_—the separator volume, m^3^. ^2^ *M*_0_, *M*—the mass of the separator before and after absorbing liquid electrolyte, kg; ^3^ *σ*—ion conductivity, mS cm^−1^; *D*—the thickness of the separator, cm; *R_b_*—the bulk resistance of the electrolyte, KΩ; *A*—the area of the separator, cm^2^; ^4^ *A*_0_, *A*—the area of the separator before and after annealing at different temperatures, respectively, m^2^.

### 2.2. Progress of Electrospun PVDF-Based LIB Separators

**Figure 2:**
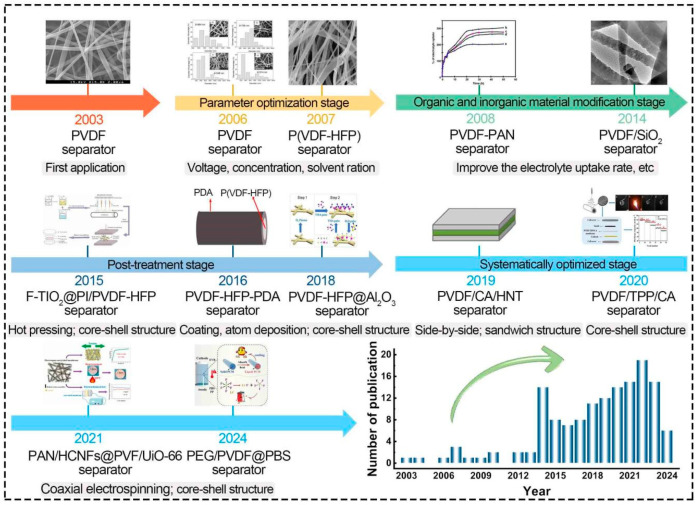
Progress of electrospun PVDF-based LIB separators. PVDF separators. Reprinted with permission from ref. [48]. Copyright Wiley-VCH 2003. Reprinted with permission from ref. [60]. Copyright 2006 Elsevier. P(VDF-HFP) separator. Reprinted with permission from ref. [31]. Copyright 2007 Elsevier. PVDF-PAN separator. Reprinted with permission from ref. [64]. Copyright 2008 Elsevier. PVDF/SiO_2_ separator. Reprinted with permission from ref. [68]. Copyright 2014 Elsevier. F-TiO_2_@PI/PVDF-HFP separator. Reprinted with permission from ref. [75]. Copyright 2015 Elsevier. PVDF-HFP-PDA separator. Reprinted with permission from ref. [79]. Copyright Elsevier 2016. PVDF-HFP@Al_2_O_3_ separator. Reprinted with permission from ref. [80]. Copyright 2018 Royal Society of Chemistry. PVDF/CA/HNT separator. Reprinted with permission from ref. [81]. Copyright 2019 Elsevier. PVDF/TPP/CA separator. Reprinted with permission from ref. [82]. Copyright 2020 Elsevier. PAN/HCNFs@PVDF/UiO-66 separator. Reprinted with permission from ref. [76]. Copyright 2021 Elsevier. PEG/PVDF@PBS separator. Reprinted with permission from ref. [78]. Copyright 2024 Elsevier.

Figure 2 depicts the research timeline of electrospun PVDF-based LIB separators. In 2003, Choi et al. [48] initially introduced the electrospun PVDF separator for LIB and suggested that it had a bright development prospect due to its excellent physicochemical properties. The ensuing development may be divided into four stages due to the current investigation, namely, parameter optimization stage, organic and inorganic material modification stage, post-treatment stage, and systematically optimized stage.

Gao et al. [43] optimized the electrospinning parameters by analyzing the relationship between PVDF separators prepared under different voltages and the cycle performance of LIBs in 2006, which helped researchers to further improve the performance of separators and LIBs by optimizing other parameters, such as PVDF concentration [22,31,60,61,62,63]. Gopalan et al. [64] modified the PVDF electrospinning solution by adding organic polyacrylonitrile in 2008, which considerably improved electrolyte uptake as well as the ionic conductivity (7.8 mS cm^−1^ at 25 °C) of separators and LIBs’ cycle performance. After that, much attention was attracted to the preparation of composite LIB separators by blending other organic and inorganic materials with PVDF [55,64,65,66,67,68,69,70], and the mechanical and thermal stability of electrospun PVDF-based separators was enhanced by post-treatment methods (such as heat treatment) [66,71,72,73,74]. Chen et al. [75] prepared the F-TiO_2_@PI/PVDF-HFP separator with remarkable mechanical properties by combining heat treatment with hot pressing for the first time, which improved LIBs’ safety.

In recent years, researchers have been interested in the electrospun PVDF-based LIB separators with thermal shutdown function [15,43,49,50,73], which systematically optimized parameters of electrospinning solution, process, and post-treatment methods [76,77]. For instance, Fu et al. [76] prepared the PAN/HCNFs@PVDF/UiO-66 separator with high strength, thermal stability, and thermal shutdown (core–shell structure) via coaxial electrospinning and hot-pressing treatment, which achieved high coulombic efficiency and superior cycle performance in LIBs. In addition, Wang et al. [78] made the PEG/PVDF@PBS (core–shell structure) separator with dual functions of cooling and thermal shutdown by a coaxial electrospinning method. As a result, research on electrospun PVDF-based LIB separators will flourish in the future.

To sum up, the electrospun PVDF-based separators need to meet the basic requirements of feature and performance parameters of LIB separators. Fortunately, controlling the electrospinning solution, process, and post-treatment methods can regulate morphology and structures to achieve the goals of optimizing the performance of separartors and LIBs [35,83,84,85].

## 3. Influencing Factors of Electrospun PVDF-Based LIB Separators

**Figure 3:**
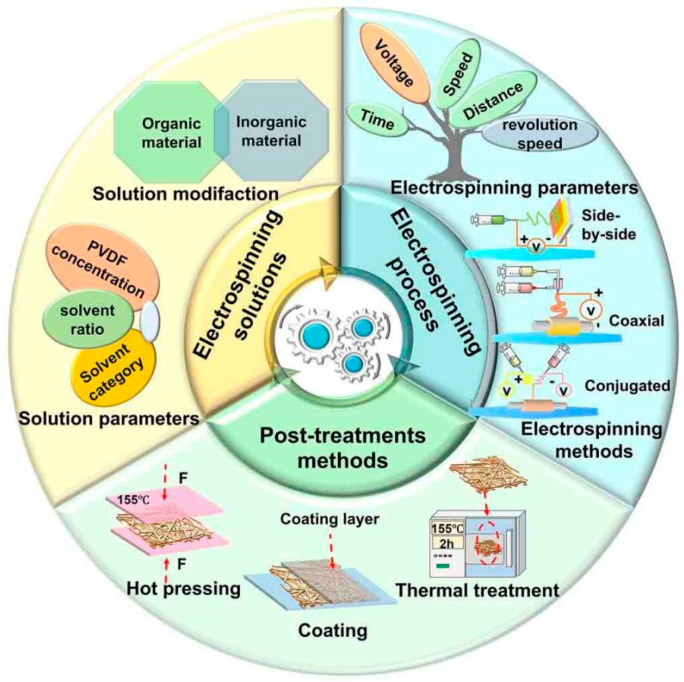
Influencing factors of electrospun PVDF-based LIB separators.

Although electrospun PVDF-based separators reach the basic specifications of LIB separators in terms of porosity, electrolyte contact angle, and thermal stability, their tensile strength and thermal stability should be further improved [47,58,86,87]. Figure 3 illustrates three main influencing parts, namely, electrospinning solution, electrospinning process, and post-processing methods.

### 3.1. Electrospinning Solution

There are two main factors (parameters and modification) that affect the electrospinning solution. Among them, the former includes solvent type, solvent ratio, and PVDF concentration [62,88]. The latter contains organic, inorganic, and synergistic modification [89,90]. The morphology, structure, and performance of electrospun PVDF-based LIB separators are affected by the solution surface tension, conductivity, and solvent volatility [91].

#### 3.1.1. Solution Parameters

1. Solvent type

The common polar solvents for PVDF are toxic N,N-dimethylformamide (DMF), N,N-dimethylacetamide (DMAc), acetone (ACET), and so on. Gee et al. [92] evaluated the effects of DMF, N-methyl pyrrolidone (NMP), and dimethyl sulfoxide (DMSO) on PVDF separator fibers. The results showed that the separators’ average fiber diameters (AFD) prepared by DMF, NMP, and DMSO were 129 ± 3.92 nm, 521 ± 313 nm, and 625 ± 113 nm, respectively. The AFD of the separator prepared by DMF was the smallest, which was about 30% smaller than that of the largest DMSO. Their porosity had little difference due to the denser fiber network and smaller average pore area obtained by DMSO. Since the electrospinning solution’s surface tension with the solvent of high saturated vapor pressure and low boiling point is small, it is extremely possible to form uniformly beadless fibers [93,94]. Nevertheless, the stretching time shortens owing to the solution volatilization and the fiber’s rapid solidification rate in the case of excess, which leads to a larger AFD [29]. The AFD and porosity of electrospun PVDF-based separators affect the safety and electrochemistry of LIBs. Uniform separators with small AFD have strong mechanical properties, which offer excellent safety. On the precondition of extraordinary mechanical properties, LIBs with high porosity separators have low internal resistance, high ionic conductivity, and power density [22,47];

2. Solvent ratio

**Figure 4:**
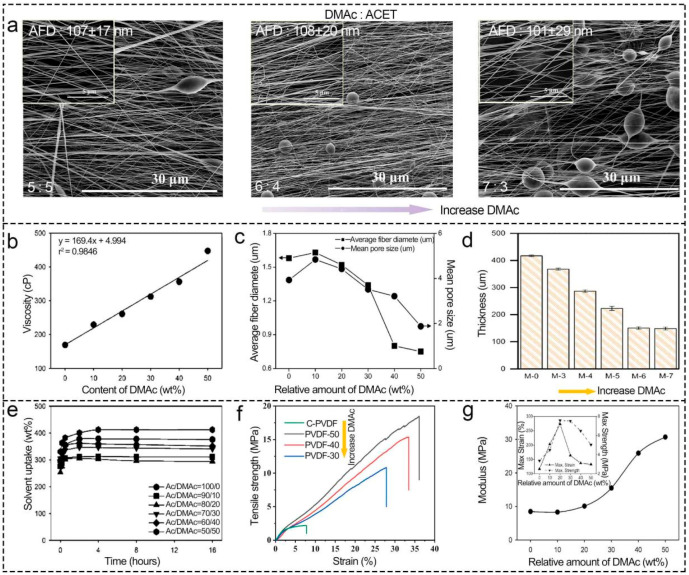
Effect of solvent ratio on morphology and properties of electrospun PVDF-based LIB separators. (**a**) morphology. Reprinted with permission from ref. [95]. Copyright 2023 Elsevier. (**b**) viscosity (**c**) AFD. Reprinted with permission from ref. [96]. Copyright 2007 American Chemical Society. (**d**) thickness. Reprinted with permission from ref. [94]. Copyright 2022 Elsevier. (**e**) electrolyte uptake. Reprinted with permission from ref. [96]. Copyright 2007 American Chemical Society. (**f**,**g**) tensile strength. Reprinted with permission from ref. [96]. Copyright 2022 American Chemical Society; Reprinted with permission from ref. [95]. Copyright 2007 Elsevier.

When the solvent type is constant, the increase in solvents with low saturated vapor pressure, large surface tension, and low volatility within a certain range results in smaller AFD, more uniform distribution of nanofibers, thinner thickness, lesser porosity, ionic conductivity, and tensile strength of separators [62]. As plotted in Figure 4a, Chen et al. [95] found that the increase in DMAc with less volatility slowed down the fiber solidification rate but promoted stretching in DMAc and ACET mixed solvent, which resulted in smaller AFD and pore size. Yet, the fiber possessed high surface tension because of the solution’s low volatility, which made them more likely to form beads and reduce the PVDF separator’s tensile strength. After that, Hu et al. [94] and Choi et al. [96] also illustrated that the increase in DMAc resulted in thinner beaded fibers, which produced thinner separators and lower ionic conductivity, in turn (Figure 4b–g). Nevertheless, Gee et al. [92] discovered that excessive DMAc would cause slow evaporation, which broke the Taylor cone and caused the subpar electrospinning process. Usually, the optimal solvent ratio needs to be altered experimentally according to the application [51,87]. Sarma et al. [97] presented a novel Electrospun Fiber Experimental Attributes Dataset (FEAD) by collating 293 data points of PVDF electrospinning from 30 experiments of the literature and their own experiments, which included solvent, polymer concentration, and applied voltage, etc. Subsequently, multi-model machine learning of regression modeling was used to obtain the target PVDF fiber size, which effectively optimized the critical parameters. Fiber diameter is a key parameter for controlling the thermal property of electrospun PVDF separators, electrolyte uptake, and electrochemical properties of LIBs [97,98]. Therefore, this work deeply reduced cost and improved the optimization efficiency of electrospinning parameters through machine learning. Based on these, we deemed that the performance of the separator could be indirectly regulated through machine learning;

3. PVDF concentration

**Figure 5:**
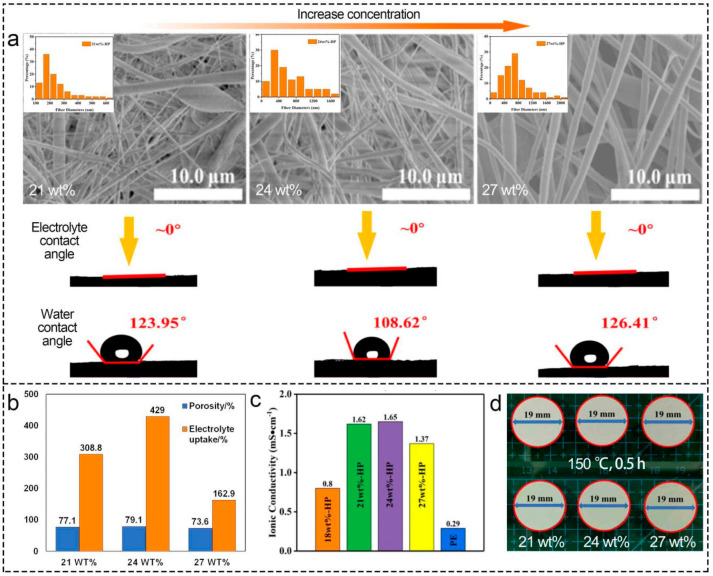
Effect of PVDF concentration (21 wt%, 24 wt%, 27 wt%) on electrospun PVDF-based LIB separators. (**a**) morphology, diameter, and contact angle. (**b**) porosity and electrolyte uptake. (**c**) ionic conductivity. (**d**) thermal stability. Reprinted with permission from ref. [100]. Copyright 2022 Wiley-VCH.

PVDF concentration is the pivotal factor that affects the morphology and fiber diameter of the electrospun separators, which positively correlates with viscosity [31]. The morphology and structure of electrospun PVDF-based separators can be optimized by increasing the PVDF concentration within a certain range, which enhances separators’ porosity, electrolyte uptake, tensile strength, and ion conductivity [22,31,62,94]. Its morphology will go through three stages: beads; beaded fibers; and uniform fibers, as the concentration increases [31,99]. Additionally, the AFD of separators increases with the addition of PVDF, while solution spinnability and separator morphology become worse due to high viscosity and tiny pull [31] when PVDF concentration exceeds a certain value. Gao et al. [100] explained that the limitation of fiber stretching was caused by the increase in solute through the electric field per unit time and found that porosity, electrolyte uptake, and ionic conductivity first increased and then decreased as the concentration of PVDF solution rose (Figure 5a). Moreover, the separator prepared with 24 wt% PVDF electrospinning solution had the highest ionic conductivity (1.65 mS cm^−1^) because of the optimal three-dimensional network structure and minimal surface roughness (Figure 5c,d). It was also presented that the thermal stability of separators, mainly determined by the material, was similar after treatment at different temperatures (Figure 5d) [100].

#### 3.1.2. Solution Modification

Electrospun PVDF separators have inferior mechanical properties compared with polyolefin separators, and their thermal stability also needs a breakthrough [14]. Solution modification has been a research hotspot by adding organic and inorganic materials to the spinning solution to address the above issues. Moreover, it can also expand the amorphous region required for Li^+^ transport and improve ionic conductivity (Table 2).

**Figure 6:**
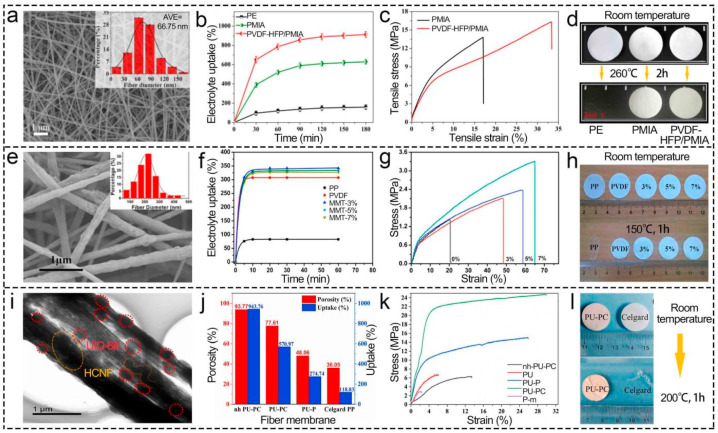
Effect of solution modification on morphology, electrolyte uptake, tensile strength, and thermal stability of electrospun PVDF-based LIB separators. (**a**–**d**) PMIA@PVDF separator. Reprinted with permission from ref. [101]. Copyright 2019 Elsevier. (**e**–**h**) PVDF/MMT separator. Reprinted with permission from ref. [109]. Copyright 2016 Elsevier. (**i**–**l**) PAN/HCNFs@PVDF/UiO-66 separator. Reprinted with permission from ref. [76]. Copyright 2021 Elsevier.

The addition of polyimide (PI), polyethylene terephthalate (PET), polymethyl methacrylate (PMIA), and other organic compounds into PVDF solution [89,101] is a simple method to effectively enhance the electrolyte uptake, tensile strength, and thermal stability of separators [64,72]. As depicted in Figure 6a–d, the LIB separators prepared by combining PMIA and PVDF-HFP had electrolyte uptake, which was as high as 913% due to the porosity growth. Furthermore, the tensile strength of the composite film was increased from about 13.50 Mpa to 16.31 Mpa, and its elongation at break rose from 17.48% to 33.75% approximately; thus, its toughness was improved simultaneously of PMIA with superior mechanical properties [101]. Furthermore, there was almost no shrinkage under heat treatment at 200 °C for 1 h, which indicated its higher thermal stability than that of commercial PE. The ionic conductivity of the organic composite separator reached up to 8.36 mS cm^−1^, prepared by Bicy et al. [46]. While the thermal stability of the aforementioned separators is better than that of commercial polyolefin separators, further work needs to be performed to reduce LIB fire accidents.

Inorganic compounds such as Al_2_O_3_ [65], SiO_2_ [35,102,103,104], and talcum (TM) [33] are usual materials for reinforcing the ionic conductivity, thermal stability, and mechanical properties of electrospun PVDF separators [36,56,69,105,106]. The interaction between hydroxyl groups in SiO_2_ and PVDF molecular chains inhibits PVDF recrystallization and reduces crystallinity, which expands the amorphous region required for Li^+^ migration [29]. Furthermore, the polar silicon hydroxyl group improves wettability and promotes Li^+^ movement, which reduces the internal resistance and increases the C-rate of LIBs [68,107]. SiO_2_ can also enhance the separators’ thermal stability and mechanical properties, which greatly reduces the probability of an internal short circuit and enhances the safety of LIBs due to lithium dendrite and separator melting [108]. Fang et al. [109] strengthened electrolyte uptake, tensile strength, and thermal stability of the separator by incorporating 5 wt% montmorillonite (MMT) into PVDF, and the electrolyte uptake was 333%, which was 8.47% higher than that of the pure PVDF separator. Its increase was mainly because of the high active surface of MMT and high affinity toward propylene carbonate/diethyl carbonate (electrolyte solution composition). Moreover, the high aspect ratio of MMT and the increase in the separator’s dielectric constant enhanced the retention of the electrolyte solution [110,111]. The presence of MMT also led to the transformation of non-polar a-phase to polar β-phase, which was beneficial to the dissolution of polar electrolytes. Due to the physical cross-linking points and inhibition of a-phase PVDF, the separator’s tensile strength was 2.39 MPa and 68.31% higher than that of the pure PVDF separator [109]. Its area was reduced by 13.5% after thermal exposure at 150 °C for 60 min because the MMT particle prevented the rapid heat transfer through the composite material, while the pure separator’s area shrinked 27%. Nevertheless, the tensile strength of 2.39 MPa should be further improved (Figure 6e–h). Most inorganic nanomaterials are poorly dispersed in PVDF and easy to fall off due to the weak interaction between them, which hinders further application of electrospun PVDF-based separators in LIBs [112]. Hence, Tiwari et al. [87] introduced the sulfonation reaction by sulfonate groups. Pan and his team [113] wrapped dopamine in nanomaterials to enhance electrostatic repulsion and reduce the interfacial energy. Nevertheless, there were still issues of uneven dispersion and easy shedding owing to the rigidity and insolubility of inorganic materials when their concentration exceeded a certain threshold [114].

The leap from inorganic or organic to composite modification realizes rigidity, heat resistance, and flexible complementarity [115,116], which offers novel ideas for electrospun high-quality PVDF-based LIB separators. Zhao et al. [117] prepared a PMIA-based gel polymer electrolyte separator containing PVDF-HFP and MMT and indicated that the addition of MMT promoted Li^+^ flow, reduced the pore size, and suppressed the “shuttle effect”, which improved the LIBs’ electrical performance and safety. The PMIA@PAN/PVDF-HFP/TiO_2_ composite separator [73] had not only flexibility but also a tensile strength of 29.7 MPa. Currently, the emerging organic–inorganic hybrid materials (MOFs [43,44,118,119] and COFs [120,121]) with a large specific surface area have attracted much attention because they could appreciably improve separator performance. The electrolyte uptake (943.76%) and tensile strength (24.77 MPa) of the composite separator were not only improved after the addition of UiO-66, but also its thermal stability (almost no shrinkage when heated at 200 °C for 1 h) was enhanced (Figure 6i–l). There are numerous kinds of materials, and the research is still in the laboratory stage.

**Table 2:** Electrospun PVDF-based LIB separators based on solution modification.

Electrospinning Solution	Thickness /μm	Diameter /nm	Porosity /%	Electrolyte Uptake /%	Ionic Conductivity /(mS cm^−1^)	Tensile Strength /MPa	Thermal Stability	Ref.
PVDF-HFP	-	150–250	80	340	1.29	6.5		[122]
PVDF-HFP/PMIA	-	66.75	93.75	913	1.20	16.31	Shrinkage rate less than PE at 240 °C for 2 h	[101]
PAN@PVDF-HFP	-	-	-	-	1.20	45.8	No significant shrinkage at 160 °C	[123]
PVDF/PDA-CE	80	140	73 ± 2	438 ± 29	2.77	10.48	No significant shrinkage at 160 °C for 1 h	[124]
PVDF-HFP@PI	22	-	80	800	1.79	13.3	No significant shrinkage at 140 °C for 0.5 h	[77]
PVDF/IL[Emim][TFSI]	-	180	72	356	2.88	>1	Thermal decomposition temperature is 458 °C	[125]
P(VDF-TrFE/PEO/LIGC	-	910	86	>440	7.04	6.1	-	[15]
PVDF/MMT	58	214	84.08	333	4.2	2.39	Shrinkage rate is 14.6% at 150 °C for 1 h	[109]
SiO_2_/PVDF-HFP	45	130–450	89.7	483	-	5	No significant shrinkage at 200 °C for 0.3 h	[126]
Sb_2_O_3_/PVDF-CTFE	42	300–400	72	356	2.88	13.5	No significant shrinkage at 160 °C for 2 h	[127]
MMT/PVDF-HFP-PMIA	-	277.2	90.21	1027	2.41	25.59	No significant shrinkage at 220 °C	[117]

### 3.2. Electrospinning Process

There are two main factors that affect the morphology, structures, and performance of PVDF-based LIB separators during the electrospinning process. The first is the electrospinning parameters, which include time, voltage, injection speed, distance, and collector speed. The second is electrospinning methods, such as layer-by-layer with single-needle, coaxial, and conjugated methods.

#### 3.2.1. Electrospinning Parameters

Electrospinning time is positively correlated with the thickness of PVDF separators [128], while pore size and porosity have little difference [46,129]. The thickness of separators cannot be precisely controlled during preparation because fibers are not fully deposited on the collector; therefore, they can only be estimated by spinning time, etc.

The relationship between applied voltage and separators’ AFD remains controversial [29]. Some researchers demonstrated that the higher the voltage, the smaller the AFD; the reason was that the jet velocity and tensile force became greater when the electrostatic force increased [22,60]. Others indicated that higher voltage led to more solutions being ejected and the growth of separators’ AFD [31,62,130]. It had also been discovered that AFD first increased and then decreased as the voltage grew [97]. Usually, the smaller the AFD with the larger specific surface area, the higher the tensile strength [19,40].

More solution passes through the electric field per unit time, and the constant electrostatic force and smaller tensile force on the droplet when the injection speed increases, which leads to the increase in AFD [29,62]. However, the electrospinning did not proceed successfully when the bolus injection speed was extremely slow [97].

With the increase in electrospinning distance, the AFD tends to decrease because of the longer stretching stroke, while it may also increase due to an increase in field strength. Since the former plays a dominant role, the AFD decreases integrally. When the distance is too large, the fiber is obviously prone to drifting [97]. The electric field strength is determined by electrospinning distance and applied voltage, as well as its effect on the PVDF β-phase, and the crystallinity of the separator is mixed. The latest research illustrated that the β-phase and crystallinity appeared to have an inflection point on both sides of the opposite rule of change when the field strength rose [29].

The collector speed was positively correlated with the β-phase content of the PVDF [29]. With the increase in rotating speed, the AFD of separators, orientation along the rotating direction, wettability, mechanical properties, ionic conductivity, and battery cycle performance were improved [131,132,133]. The faster speed endowed more tensile force on fibers in a direction tangent with drum rotation, which tended to lead to fiber orientation [29]. Zheng et al. [134] observed that the orientation and tensile strength of 0°PAN/PVDF/90°PAN separators were the best at 900 r min^−1^ compared with those at 300, 600, and 900 r min^−1^. The tensile strength of the PVDF-HNT separators increased from 24.1 MPa (random) to 32 MPa (2000 r min^−1^) [135]. Liu et al. [123] demonstrated that the tensile strength of the PAN@PVDF-HFP separator prepared at 2800 r min^−1^ was greater than that of the random separators, but it had the mechanical anisotropy, namely, the high tensile strength along the orientation direction. Thus, improving mechanical properties in both directions is a serious problem that needs to be solved urgently. In addition to a fixed speed, Xing et al. [45] also researched that the orientation gradient separator prepared by the gradient speed was higher than that of the non-gradient separator in porosity, wettability, ion transport, and tensile strength. This may be the fact that different orientations formed larger pore sizes and increased the contact points of the upper and lower fibers, which provided mechanical support [45]. However, there is little research on the preparation of electrospun PVDF-based gradient separators and their application in the LIBs. In terms of the ion conduction mechanism, Liu et al. [123] explained that the regular channels inside the directional fibers reduced the Li^+^ diffusion time. It was also proved that the composite separators at a high speed of 3500 r min^−1^ had unprecedented cycle performance in sodium–metal batteries [136], while the electrospun PVDF-based separators prepared at high speed were rarely reported in the LIBs field.

Electrospinning parameters and solution parameters have an interactive influence on the PVDF-based separator morphology and LIBs performance; therefore, it is necessary to explore the dominant factor and balance these parameters. PVDF concentration is the most critical factor that affects morphology and structures [31,62]. As for the research on balancing parameters, Essam [137] optimized the PVDF concentration, voltage, flow rate, and other parameters using the control variable method. Gee et al. [92] designed orthogonal experiments with four factors and three levels (solvent ratio, electrospinning distance, applied voltage, and injection speed) and proposed that solvent ratio had the greatest effect on the PVDF β-phase conversion without an investigation of the LIB’s performance.

#### 3.2.2. Electrospinning Methods

Various electrospinning methods, such as layer-by-layer with single-needle, coaxial, and conjugated methods, can prepare sandwich, core–shell, hollow, and other structures. These structures improved the porosity, ionic conductivity, and thermal stability of the separators and also achieved other functions, which optimized the performance of LIBs [67,124,138,139,140]. The specific parameter values are displayed in Table 3.

**Figure 7:**
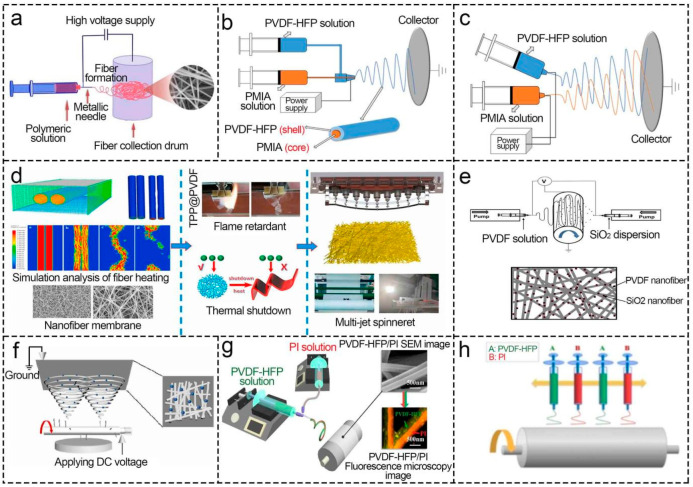
Effect of electrospinning methods on PVDF-based LIB separators. (**a**) PA6/PVDF-HFP/PA6 (general syringe, layer-by-layer). Reprinted with permission from ref. [141]. Copyright 2020 MDPI, Basel. (**b**) PMIA@PVDF-HFP (coaxial electrospinning) and (**c**) PMIA/PVDF-HFP (simultaneous electrospinning). Reprinted with permission from ref. [142]. Copyright 2020 Elsevier. (**d**) TPP@PVDF (coaxial electrospinning). Reprinted with permission from ref. [49]. Copyright 2023 Elsevier. (**e**) SiO_2_/PVDF (conjugated electrospinning). Reprinted with permission from ref. [107]. Copyright 2014 Elsevier. (**f**) SiO_2_/PVDF (needleless electrospinning). Reprinted with permission from ref. [147]. Copyright 2022 Elsevier. (**g**) PVDF-HFP/PI (side-by-side electrospinning) and (**h**) PVDF-HFP + PI (four-needle cross-electrospinning). Reprinted with permission from ref. [148]. Copyright 2020 Elsevier.

The separator is expected to achieve a thermal shutdown function when the materials of each layer are different. The PA6/PVDF-HFP/PA6 separator with sandwich structure prepared layer by layer is presented in Figure 7a [141]. The inner layer melts, and the micropore closes as the temperature rises, which makes the Li^+^ stay and the battery go out of service. The core, as the skeleton, maintains the morphology and mechanical strength, and the shell accommodates electrolytes and provides high ionic conductivity in the core–shell structure separators prepared by coaxial electrospinning [78,89]. For example, the core PAN supplied mechanical support to LIBs, and the shell PVDF housed the electrolyte in the PAN/PVDF separator [138]. Chen et al. [142] discussed the influence of different electrospinning methods on the structures of separators and battery performance based on PMIA and PVDF-HFP (Figure 7b,c), in which the separators prepared by coaxial electrospinning had more uniform fiber diameters, smaller electrolyte contact angles, and greater ionic conductivity and LIBs’ cycle stability [143]. Moreover, coaxial electrospinning enabled the separators to achieve dual functions of thermal shutdown and flame retardant, where the core-to-shell ratio was critical [89]. If there is much shell content, the flame retardant effect is poor, and the thermal shutdown function cannot be realized owing to the internal flame retardant not being released. Based on this, in 2023, Zheng et al. [49] optimized the TPP@PVDF separator core-to-shell ratio for the first time by machine learning (Figure 7d), but it still needs to be extended to other core–shell separators. LIB separators with reversible thermal shutdown suspended work at a certain temperature (e.g., PVP@TiO_2_ at 60 °C [144]) and resumed Li^+^ transmission at room temperature, which was economically practical [145]. Nevertheless, the thermal shutdown of most electrospun PVDF-based LIB separators was not reversible. Therefore, it was possible to realize reversibility through the application of shape-memory polymers [146] and phase-change materials [145].

The conjugated electrospinning is also an approach to preparing safe and high-performance LIB separators. Figure 7e presents a schematic diagram of the SiO_2_/PVDF composite separator, which not only had the thermal shutdown function but also enhanced porosity, electrolyte uptake, ionic conductivity, and electrochemical stability window after introducing SiO_2_ [107]. However, its thickness with complex structures needed to be thinned. Various methods, such as needleless electrospinning [147], side-by-side with a double-needle, and four-needle crossover [148], have gradually emerged to improve productivity and expand the separator structures (Figure 7f–h). The novel biomimetic structures, including a silkworm cocoon structure with high porosity, can also be explored in the future [149].

**Table 3:** PVDF-based LIB separators prepared by different electrospinning methods.

Separators	Electrospinning Methods	Thickness /μm	Diameter /nm	Porosity /%	Electrolyte Uptake /%	Ionic Conductivity/(mS cm^−1^)	Tensile Strength /MPa	Thermal Stability	Ref.
PA6/PVDF-HFP/PA6	single-needle, layer-by-layer	60	200–500	90.35	230	4.2	17.11	Thermal shutdown function at 145 °C No significant shrinkage at 230 °C	[141]
TPP@PVDF	Coaxial electrospinning	-	355.42	85.13	339.72	1.829	-	Thermal shutdown function at 177 °C	[49]
PAN/PVDF	Coaxial electrospinning	60	249.3	81.61	-	1.62	3.6	No significant shrinkage at 170 °C for 1 h	[138]
SiO_2_/PVDF	Conjugated electrospinning	-	-	70 ± 6	370 ± 9	2.6 ± 0.3	13	No significant shrinkage at 150 °C for 0.5 h	[107]
SiO_2_/PVDF	Needleless electrospinning	60	-	134.5	541.6	1.43, 25 °C	1.3	No significant shrinkage at 140 °C	[147]
M-PAN/PVDF-HFP	Side-by-side electrospinning	-	372 ± 41	82.09	553.23	2.81	14.36	Shrinkage rate is 30% at 200 °C for 0.5 h	[50]

### 3.3. Post-Treatment Methods

Post-treatment methods, such as heat treatment, coating, and hot pressing, regulate the morphology, fiber diameter, porosity of separators, and so on, which indirectly determine the mechanical properties, ionic conductivity, and thermal shutdown function of the separators and LIBs [150]. The specific parameter values are presented in Table 4.

#### 3.3.1. Heat Treatment

**Figure 8:**
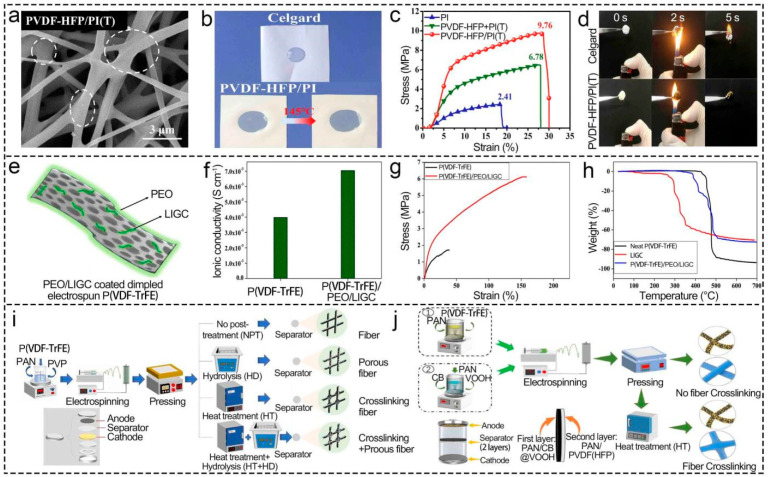
Effect of post-treatment methods on morphology, ionic conductivity, tensile strength, and thermal stability of electrospun PVDF-based LIB separators. (**a**–**d**) thermal treatment. Reprinted with permission from ref. [148]. Copyright 2020 Elsevier. (**e**–**h**) coating treatment. Reprinted with permission from ref. [15]. Copyright 2022 Elsevier. (**i**,**j**) hot-pressing treatment. Reprinted with permission from ref. [51]. Copyright 2022 Elsevier; Reprinted with permission from ref. [155]. Copyright 2023 Elsevier.

Heat treatment made the AFD larger because of the expanded fiber [71]. Although the porosity, electrolyte uptake, and ionic conductivity of the separator were all exceeded by those of the polyolefin separators, they only slightly decreased [71,122]. The electrolyte uptake determined by pore size, porosity, and specific surface area was the most critical factor that affected the ionic conductivity and played a leading role in the electrochemical performance of the LIBs. The increase in fiber diameter and the adhesion between fibers led to a low porosity of the separator after heat treatment, which caused the low ionic conductivity result from the decrease in electrolyte absorption rate and high bulk resistance [71]. Furthermore, the mechanical properties of electrospun PVDF-based separators are enhanced, which results from the increased interaction force of fibers and crystallinity due to the fiber cross-linking and the bonding point, as well as the rearrangement orientation of the PVDF molecular chains during annealing and recrystallization, respectively [60,71,151]. Ding et al. [122] treated PVDF-HFP separators at different low temperatures and found that the fiber diameter and fracture strain increased significantly with the rise in temperature, coupled with the improvement in ionic conductivity (increased by 2.78 mS cm^−1^ at 75 °C compared with that at 25 °C). As illustrated in Figure 8a–d, the tensile strength of the PVDF-HFP/PI separator rose from 7.08 MPa to 9.76 MPa after treatment at 140 °C for 20 min [148]. Since the excessively high heat treatment temperature easily caused serious melting of fibers and destroyed the high porosity’s advantage of electrospinning, the heat treatment temperature was usually near the separator melting point to balance the electrical and mechanical properties [93].

#### 3.3.2. Coating

Coating flame-retardant materials reinforced separators’ safety [26,151,152,153]. The ethylene oxide/wood fiber-coated P(VDF-TrFE) brought the problem of no self-shutdown function to a close (Figure 8e–h) [15]. Wu et al. [19] coated Al_2_O_3_ on both sides of the electrospun PVDF separator, which improved its thermal stability (only shrinking by about 2% at 140 °C) and maintained high ionic conductivity (2.23 mS cm^−1^). However, the porosity of the separator was reduced (55.8%), which destroyed the porous structure and reduced the specific energy of the LIBs. Coating materials with good wettability can enhance the battery’s electrical performance [154]. The electrospun PVDF separators coated with the PDA maintained their original structures and thickness, which promoted a Li^+^ transfer [120]. Guo et al. [50] selected the self-polymerization of catechol and TEPA to simulate the modification process of PDA and successfully prepared the M-PAN/PVDF-HFP separator with a high ionic conductivity (2.81 mS cm^−1^) due to the high cost of PDA.

#### 3.3.3. Hot-Pressing Treatment

Co-processing is also crucial compared to the single post-processing method. The separator with thinner, more complete, and higher mechanical properties was prepared by the hot-pressing process (Figure 8i,j) [26], which was obvious that the pressing made the fiber tighter and the physical bonding stronger. Heat treatment made the molecular chain of the fiber adhere, which hindered the debonding of the fiber and the slip of the molecular chain [51,155]. The tensile strength of the PAN/CB/VOOH-PAN/PVDF (HFP) separator was up to 20.8 MPa after pressing at 25 MPa for 20 min and then a heat treatment at 155 °C for 30 min [51]. The tensile strength of the PAN/PVDF-HFP/PVP separator was increased by three times; the fibers’ bonding point was bonded by the hot-pressing process [155], and the PAN/HCNFs@PVDF/Uio-66 separator with core–shell structure had a higher tensile strength, porosity, and dimensional integrity at 200 °C after the hot-pressing treatment [76].

In short, the electrospinning solution, process, and post-treatment methods have a significant influence on the morphology, structures, and performance of the electrospun PVDF-based LIB separators and can be optimized according to their influence law.

**Table 4:** Electrospun PVDF-based LIB separators prepared by different post-treatment methods.

Separators	Electrospinning Methods	Post- Treatment Methods	Thickness /μm	Diameter /nm	Porosity /%	Electrolyte Uptake /%	Ionic Conductivity/(mS cm^−1^)	Tensile Strength/MPa	Thermal Stability	Ref.
PVDF-24 wt%	General syringe	Thermal treatment at 80 °C for 2 h	70	237.4–626.8	79.1	429	1.65	-	No significant shrinkage at 150 °C for 0.5 h	[100]
PVDF-HFP/PI	Side-by-side electrospinning	Thermal treatment at 145 °C for 20 min	23	177.9	85.9	483.5	1.78	9.76	No significant shrinkage at 200 °C for 0.5 h	[144]
PAN/PVDF-HFP/PVP	General syringe	Thermal treatment at 170 °C for 0.5 h	-	-	74.5	605.8	1.97	22.13	No significant shrinkage at 200 °C	[155]
PMIA@PAN/PVDF-HFP/TiO_2_	Coaxial electrospinning	Thermal treatment at 170 °C	-	-	48.2	207.3	1.36	29.7	No significant shrinkage at 220 °C for 1 h	[73]
PAN/CB/VOOH-PAN/PVDF-(HFP)	General syringe	Pressing under 25 MPa for 1/3 h and then heat treated at 155 °C for 0.5 h	-	10–90	70.7	510.4	2.81	20.8	No significant shrinkage at 250 °C	[51]
PAN/HCNFs@PVDF/UiO-66	Coaxial electrospinning	Hot pressing at 120 °C under 10 MPa for 2 h	25	489.6	77.61	570.97	1.59	24.77	No significant shrinkage at 200 °C for 1 h	[76]
0°PAN/PVDF/90°PAN	General syringe, layer-by-layer	Hot pressing at 35 °C under 2 MPa for 60 s	46	210	85.64	-	-	10.33	Slight folds at 180 °C for 0.5 h	[134]

## 4. Conclusions and Outlook

Electrospun PVDF-based separators are expected to achieve the goal of safe and high-performance LIBs due to their high porosity, excellent wettability, and thermal stability. This paper systematically summarized the process and key parameter requirements of the electrospun PVDF-based LIB separators. More importantly, the effects of the electrospinning solution, process, and post-treatment methods on the morphology, structure, and properties of the electrospun PVDF-based LIB separators were also reviewed, which covered the entire preparation process of the electrospun PVDF-based separators. Although remarkable research has been obtained on the electrospun PVDF LIB separators, there are still some limitations and challenges:

(1) Electrospinning solution. The development and application of green solvents are critical to the sustainable development of electrospun PVDF-based LIB separators due to the toxic nature of most solvents. Furthermore, the inferior dispersion resulting from inorganic nanomaterials can be alleviated by washing with organic solvents of low-surface tension, such as anhydrous ethanol, adding surfactants during the preparation of fillers, and functionalizing nanomaterial. In addition, emerging materials such as MOFs and COFs with large specific surface areas and high-strength carbon fibers should be further researched;
(2) Electrospinning process. Traditional orthogonal experiments are inefficient in terms of optimizing electrospinning parameters; thus, it may be possible to combine machine learning to efficiently predict the setting of electrospinning conditions in the future, and the interaction research of gradient speed and other factors on the morphology and properties of electrospun PVDF-based LIB separators will be more systematic;
(3) Post-processing methods. The inorganic material coating will cause the risk of clogging separator pores, which will be alleviated by electrophoretic coating. Moreover, although separators theoretically meet the operating temperature of the LIBs, the internal temperature rises rapidly and continuously when the thermal runaway of the LIBs occurs, which exceeds the withstand temperature of the separators. Therefore, it is pivotal to continuously improve the separators’ thermal stability to ensure the safety of the LIBs.

In summary, the electrospun PVDF-based LIB separators have broad development space and application prospects, which will promote their further application and development of the LIBs and the achievement of safety and carbon neutrality.

## Data Availability

Data are contained within the article.

## Appendix A

**Table A1:** The list of abbreviations with their explanations.

Number	Abbreviation	Explanation	Number	Abbreviation	Explanation
1	PVDF	Poly(vinylidene fluoride)	23	AFD	Average fiber diameter
2	LIBs	Lithium-ion batteries	24	PI	Polyimide
3	Li^+^	Lithium-ions	25	PET	Polyethylene terephthalate
4	PP	Polypropylene	26	PMIA	Polymethyl methacrylate
5	PE	Polyethylene	27	TM	Talcum
6	PAN	Polyacrylonitrile	28	MMT	Montmorillonite
7	HCNFs	Helical carbon nanofibers	29	MOFs	A metal-organic framework
8	UiO-66	A Zr-based metal-organic framework	30	COFs	A novel hollow tube covalent organic frameworks
9	PEG	Polyethylene glycol	31	CE	4′-aminobenzo-15-crown-5
10	PBS	Polybutylene succinate	32	IL	Ionic liquids
11	PVDF-HFP	Polyvinylidene fluoride-hexafluoropropylene	33	[Emim][TFSI]	1-ethyl-3-methylimidazolium bis(trifluoromethylsulfonyl)imide
12	SiO_2_	Silicon dioxide	34	P(VDF-TrFE)	Poly(vinylidene fluoride-trifluoroethylene
13	F-TiO_2_	Functionalized TiO_2_	35	PEO	Polyethylene oxide
14	PDA	Polydopamine	36	LIGC-[15]	Lignocellulose
15	Al_2_O_3_	Aluminium oxide	37	Sb_2_O_3_	Antimony trioxide
16	CA	Cellulose acetate	38	PVDF-CTFE	Poly(vinylidene fluoride-co-chlorotrifluoroethylene)
17	HNT	Halloysite nanotube	39	TPP	Triphenyl phosphate
18	DMF	N,N-dimethylformamide	40	PA6	Nylon 6
19	DMAc	N,N-dimethylacetamide	41	CB	Carbon black
20	ACET	Acetone	42	VOOH	The adsorption-catalysis material synthesized hydrothermally
21	NMP	N-methyl pyrrolidone	43	PVP	Polyvinyl pyrrolidone
22	DMSO	Dimethyl sulfoxide	/		
